# Association of Fluid Accumulation with Clinical Outcomes in Critically Ill Children with Severe Sepsis

**DOI:** 10.1371/journal.pone.0160093

**Published:** 2016-07-28

**Authors:** Jiao Chen, Xiaozhong Li, Zhenjiang Bai, Fang Fang, Jun Hua, Ying Li, Jian Pan, Jian Wang, Xing Feng, Yanhong Li

**Affiliations:** 1 Pediatric Intensive Care Unit, Children’s Hospital of Soochow University, Suzhou, JiangSu province, China; 2 Department of Nephrology, Children’s Hospital of Soochow University, Suzhou, JiangSu province, China; 3 Institute of Pediatric Research, Children’s Hospital of Soochow University, Suzhou, JiangSu province, China; 4 Department of Neonatology, Children’s Hospital of Soochow University, Suzhou, JiangSu province, China; Bambino Gesù Children's Hospital, ITALY

## Abstract

**Objective:**

To evaluate whether early and acquired daily fluid overload (FO), as well as fluctuations in fluid accumulation, were associated with adverse outcomes in critically ill children with severe sepsis.

**Methods:**

This study enrolled 202 children in a pediatric intensive care unit (PICU) with severe sepsis. Early fluid overload was defined as ≥5% fluid accumulation occurring in the first 24 hours of PICU admission. The maximum daily fluid accumulation ≥5% occurring during the next 6 days in patients with at least 48 hours of PICU stay was defined as PICU-acquired daily fluid overload. The fluctuation in fluid accumulation was calculated as the difference between the maximum and the minimum daily fluid accumulation obtained during the first 7 days after admission.

**Results:**

Of the 202 patients, 61 (30.2%) died during PICU stay. Among all patients, 41 (20.3%) experienced early fluid overload, including 9 with a FO ≥10%. Among patients with at least 48 hours of PICU stay (n = 154), 36 (23.4%) developed PICU-acquired daily fluid overload, including 2 with a FO ≥10%. Both early fluid overload (AOR = 1.20; 95% CI 1.08–1.33; P = 0.001; n = 202) and PICU-acquired daily fluid overload (AOR = 5.47 per log increase; 95% CI 1.15–25.96; P = 0.032; n = 154) were independent risk factors associated with mortality after adjusting for age, illness severity, etc. However, fluctuations in fluid accumulation were not associated with mortality after adjustment. Length of PICU stay increased with greater fluctuations in fluid accumulation in all patients with at least 48 hours of PICU stay (FO <5%, 5%-10% vs. ≥10%: 4 [3–8], 7 [4–11] vs. 10 [6–16] days; P <0.001; n = 154) and in survivors (4 [3–8], 7 [5–11] vs. 10 [5–15] days; P <0.001; n = 121). Early fluid overload achieved an area under-the-receiver-operating-characteristic curve of 0.74 (95% CI 0.65–0.82; P <0.001; n = 202) for predicting mortality in patients with severe sepsis, with a sensitivity of 67.2% and a specificity of 80.1% at the optimal cut-off value of 2.65%.

**Conclusions:**

Both early and acquired daily fluid overload were independently associated with PICU mortality in children with severe sepsis.

## Introduction

Sepsis remains a leading cause of death among children [1,2]. Fluid resuscitation is integral to children with severe sepsis and septic shock because the major pathophysiologic changes in patients with septic shock include distributive shock and cardiogenic shock [3–5]. The implementation of early goal-directed therapy (EGDT), as a guide for acute resuscitation in severe sepsis and septic shock, has been shown to reduce mortality and both hospital and intensive care unit (ICU) length of stay in adults [4,6,7]. However, strong evidence is lacking to confirm that EGDT, which consists of early and aggressive fluid resuscitation, has beneficial effects on the clinical outcomes of pediatric patients [8–10].

Fluid administered to critically ill children during the resuscitation phase has the potential to accumulate, which might lead to subsequent fluid overload associated with adverse outcomes [11–14]. Although inconsistencies exist [15,16], the majority of fluid overload studies have reported that fluid overload is associated with an increased risk of mortality in various pediatric populations [11,16–20]. Our previous study conducted in 370 critically ill children admitted to the pediatric ICU (PICU), including both medical and surgical patients, showed that early fluid overload developed during the first 24 hours after PICU admission was associated with increased risk of mortality [21].

In adult patients with severe sepsis and septic shock, persistent fluid overload is common and associated with increased hospital mortality [22–24]. In critically ill children with sepsis or shock including septic and non-septic shock states, the presence and severity of early fluid accumulation developed during the initial 3 days after admission are associated with PICU mortality [11]. However, studies establishing the link between fluid overload and outcomes are lacking in children with severe sepsis. The aim of this study was to evaluate the association of fluid accumulation with clinical outcomes in children with severe sepsis. We analyzed whether a positive fluid balance developed in the first 24 hours of PICU admission and during the next 6 days, as well as fluctuations in fluid accumulation during the first 7 days of PICU stay, were associated with adverse outcomes in critically ill children admitted to the PICU with severe sepsis.

## Patients and Methods

### Cohorts, setting, and data collection

All children with severe sepsis who were admitted to the 20-bed medical/surgical PICU in a single standing children’s hospital in an urban setting from January 2011 to March 2015 were eligible for inclusion in the study. The selection of the time period was arbitrary. The exclusion criteria were age of less than 1 month or more than 18 years, unexpected discharge from the PICU or transfer to another hospital, and incomplete data on fluid balance. The Institutional Review Board of the Children’s Hospital of Soochow University approved the study, and all clinical investigations were conducted according to the principles expressed in the Declaration of Helsinki. Informed consent was not required because all data were collected retrospectively. Patient records were anonymized and de-identified prior to analysis.

Clinical and laboratory data were collected on the day of admission and included age, gender, and admission diagnosis. Clinical status as defined by illness severity; therapeutic interventions and medication management, including antibiotics, inotropes/vasopressors, steroids, mechanical ventilation, diuretics, and renal replacement therapy (hemofiltration); and comorbidities were recorded daily until hospital discharge or death. The inotropic score obtained during each patient’s PICU stay was adapted from Wernovsky et al. [25] and calculated retrospectively based on the following formula: Inotropic score = dopamine dose (μg/kg/min) + dobutamine dose (μg/kg/min) + 100 x epinephrine dose (μg/kg/min) + 100 x norepinephrine dose (μg/kg/min), in accordance with previous studies [26,27]. In addition, the use of antibiotics, inotropes, and steroids prior to PICU admission were recorded.

### Pediatric index of mortality 2 score

The pediatric index of mortality 2 (PIM2) score, which was calculated based on physiological parameters collected in the first hour after PICU admission, according to methods described in the original study, was used as a measure to assess illness severity and to predict probability of death [28].

### Fluid assessment

The presence, severity, and fluctuation of fluid overload were evaluated during a period defined as the entire length of a PICU stay if it was <7 days or as the initial 7 days of a PICU stay if it was ≥7 days. Daily fluid accumulation was calculated for each patient, according to the following formula: Percentage of fluid accumulation = [fluid intake (L)—fluid output (L)] / PICU admission weight (Kg) × 100% [29–31]. Daily fluid intake included intravenous maintenance fluids, parenteral and enteral nutrition, blood products, and medications. Daily fluid output included urine, stool, blood loss, gastric aspirate, and losses from other body cavities. Insensible losses were not calculated. Fluid intake and output prior to PICU admission were not available due to practical constraints.

A percentage fluid accumulation ≥5% was defined as fluid overload (FO%), which has been used in similar pediatric studies [17,32] as well as in our previous study [21]. Fluid overload occurring in the first 24 hours of PICU admission was defined as early fluid overload. A maximum daily fluid accumulation of ≥5% occurring during the next 6 days was considered to be PICU-acquired daily fluid overload. Fluctuation in fluid accumulation was calculated as the difference between the maximum and the minimum daily fluid accumulation obtained during the first 7 days after PICU admission (Max—Min). The maximum daily fluid accumulation was the most positive 24-hour fluid balance, and the minimum daily fluid accumulation was the most negative 24-hour fluid balance during the first 7 days after PICU admission. When the minimum daily fluid accumulation was negative 24-hour fluid balance, the fluctuation in fluid accumulation = [Max—(-Min)]. All patients were analyzed for early fluid overload. PICU-acquired daily fluid overload and fluctuations in fluid accumulation during the first 7 days after PICU admission were analyzed in patients who had at least 48 hours of PICU stay.

In addition, the degree of fluctuation in fluid accumulation was stratified into 3 categories: <5%, 5%-10%, and ≥10%. We explored outcomes among patients with fluctuations in fluid accumulation ≥10% above baseline. Net fluid accumulation, an expression of the cumulative FO% from the day of PICU admission to the given study day was also calculated using a formula listed in the Supporting Information (S1 Text).

### Fluid therapy

Protocolized fluid therapy was not utilized. Fluid administration and nutritional support route were determined by the treating physicians. Patients with acute kidney injury (AKI) or fluid accumulation ≥10% were considered to need renal replacement therapy (RRT).

### Definition and diagnostic criteria

Sepsis was defined as the presence (probable or documented) of infection together with systemic manifestations of infection. The diagnoses of severe sepsis and septic shock were based on the International Guidelines from the Surviving Sepsis Campaign [4]. The diagnosis of AKI was based on the AKI network criteria [33]. Baseline creatinine (Cr) was defined as the first Cr measurement during hospitalization. For patients with elevated serum Cr (>106.1 umol/L) at PICU admission, the lowest Cr value within 2 weeks during hospitalization was considered as the baseline Cr, in accordance with previous studies [21,34]. The diagnosis of ALI was based on the Consensus Recommendations from the Pediatric Acute Lung Injury Consensus Conference [35]. Multi-organ dysfunction syndrome (MODS) was defined as the simultaneous occurrence of dysfunction in two or more organs according to criteria modified from the international pediatric sepsis consensus conference [36].

### Clinical outcomes

PICU mortality was designed as the primary outcome and defined as all-cause mortality occurring during the PICU stay, including deaths resulting from withdrawal of therapy. The secondary clinical outcomes were the incidences of AKI, ALI, shock, DIC, and MODS; the need for and duration of mechanical ventilation; and the length of the PICU stay.

### Statistical analysis

Statistical software (version 20.1, SPSS) was used for data analyses. Assumptions of normality and homogeneity of variance were first checked. The Mann–Whitney U test was used to determine differences in continuous variables between two groups, and the Kruskal-Wallis H test was used to analyze differences among groups. The chi-square test or Fisher’s exact test were used to compare differences in categorical variables between groups. Spearman’s analysis was used to examine correlations. Univariate regression analysis was performed to calculate odds ratio (OR) with 95% confidence interval (CI) to assess the association of fluid overload with clinical outcomes. Multivariable logistic regression models were performed to identify independent variables associated with outcomes. Model fit was assessed by the Hosmer-Lemeshow goodness-of-fit test. A non-significant value (P >0.05) for the Hosmer-Lemeshow goodness-of-fit test suggested the absence of a biased fit. Variance inflation factor and tolerance values were calculated to determine multicollinearity. For PICU-acquired daily fluid overload analysis, continuous variables entered into the multivariable analysis were log-transformed to fit model assumptions. The sensitivity and specificity were calculated at the optimal cut-off values using Sigma Plot 10.0 software. Analysis of the area under-the-receiver-operating-characteristic curve (AUC) was used to assess the predictive strength, and the nonparametric method of Delong was performed to compare differences between AUCs.

## Results

### Patient characteristics

In total, 222 children were admitted to the PICU with severe sepsis during the study period. Among these, 20 were excluded: 1 patient was older than 18 years, 7 were unexpectedly discharged due to economic reasons, 3 were transferred to another hospital, and 9 had incomplete data on fluid balance. The remaining 202 patients made up the study cohort. The leading cause of PICU admission was respiratory diseases (40.1%), followed by gastrointestinal diseases (21.8%) and neurologic diseases (19.3%). There was no significant difference in the admission diagnoses between the survivors and the non-survivors (P = 0.703).

Severe sepsis was diagnosed in 200 (99.0%) patients on the first day after admission and in 2 (1.0%) patients on the second day after admission. Among all the patients, gram-positive bacteria were isolated from 60 (29.7%) patients, including from the blood in 44 patients, from the cerebrospinal fluid in 7 patients, from the sputum in 6 patients, from the bone marrow in 1 patient, from pleural effusion in 1 patient, and from urine culture in 1 patient. Gram-negative bacteria were isolated from 34 (16.8%) patients, including from the blood in 27 patients, from the sputum in 4 patients, from the cerebrospinal fluid in 2 patients, and from pleural effusion in 1 patient. Mycoplasmas were isolated from 8 (4.0%) patients, including from the blood in 3 patients and from sputum cultures in 5 patients. One hundred (49.5%) of the patients were diagnosed with culture-negative sepsis. In addition, among all the patients, viral DNA was isolated from the blood of 10 (5.0%) patients, and anti-virus IgM antibodies were detected in 5 (2.5%) patients. There were no patients with positive fungal culture.

The PICU mortality rate of the whole cohort was 30.2% (61/202). The median length of PICU stay was 4 (IQR 2–8) days. Among all patients, 68.3% were infants. There was a weak correlation between age and PIM2 (r = 0.197, P = 0.005) or mortality (r = 0.188, P = 0.007). AKI was diagnosed in 36 (17.8%) patients, including 2 (5.6%) classified as stage 1 on the first day after PICU admission; 8 (22.2%) classified as AKIN stage 2, 7 on the first and 1 on the fifth day after PICU admission; and 26 (72.2%) classified as stage 3, 17 on the first, 6 on the second, 1 on the third, and 2 on the fourth day after PICU admission. Among all the patients, 23 developed both AKI and fluid overload during the first 7 days after PICU admission: 8 patients were diagnosed with AKI prior to the occurrence of fluid overload, 3 patients occurred fluid overload prior to AKI diagnosis, and 12 patients were diagnosed with AKI and fluid overload on the same day.

All patients received antibiotics on the first day after admission, and 24 (11.9%) received a change in antibiotic administered based on their culture results during their PICU stay. Among the 65 patients who received inotropes during their PICU stay, 58 (89.2%) received on the first day and 7 (10.8%) received inotropes on the second day after admission. There was no significant difference when comparing the time to inotropes between the survivors and the non-survivors (P = 0.695). A comparison of the demographic and clinical characteristics between the patients with severe sepsis who did and did not survive is displayed in Table 1. In addition, there was no statistical difference in inotrope (P = 0.891), steroid (P = 0.101) or antibiotic administration (P = 0.342) before PICU admission between survivors and non-survivors.

**Table 1:** Comparison of demographic and clinical characteristics between survivors and non-survivors.

	All patients	Survivors	Non-survivors	P
	n = 202	n = 141	n = 61	
Age, years	0.5 [0.2–1.5]	0.4 [0.2–1.1]	0.9 [0.3–2.7]	0.008
Infants, n	138 (68.3)	104 (73.8)	34 (55.7)	0.011
Children, n	65 (32.0)	39 (27.7)	25 (41.0)	0.062
Body weight, kg	7.6 [5.5–10]	7.5 [5.3–10]	8 [5.7–14.3]	0.084
Male, n	107 (53.0)	74 (52.5)	33 (54.1)	0.833
PIM2	4.2 [2.1–22.0]	2.8 [2.0–8.2]	21.0 [7.5–63.8]	<0.001
Early fluid balance, during the first 24 hours after PICU admission				
Early fluid accumulation, %	0.91 [-0.44–2.82]	0.62 [-0.47–2.19]	3.00 [-0.23–5.28]	<0.001
Fluid intake, L	0.77 [0.47–1.25]	0.75 [0.45–1.11]	0.92 [0.59–1.78]	0.003
Fluid output, L	0.59 [0.36–1.05]	0.63 [0.41–1.02]	0.49 [0.17–1.19]	0.174
The maximum daily fluid balance, during the 2 to 7 day period following admission				
PICU-acquired fluid accumulation, %	3.48 [1.73–4.83]	3.27 [1.66–4.61]	4.27 [2.74–6.56]	0.054
Fluid intake, L	0.90 [0.70–1.36]	0.80 [0.70–1.27]	1.06 [0.74–1.85]	0.036
Fluid output, L	0.69 [0.50–1.00]	0.67 [0.51–0.92]	0.82 [0.46–1.13]	0.397
AKI^a^, n	36 (17.8)	18 (12.8)	18 (29.5)	0.004
Need for RRT^a^, n	40 (19.8)	20 (14.2)	20 (32.8)	0.002
ALI^a^, n	85 (42.1)	36 (25.5)	49 (80.3)	<0.001
Shock^a^, n	59 (29.2)	34 (24.1)	25 (41.0)	0.015
DIC^a^, n	25 (12.4)	7 (5.0)	18 (29.5)	<0.001
MODS^a^, n	52 (25.7)	19 (13.5)	33 (54.1)	<0.001
MV^a^, n	92 (45.5)	40 (28.4)	52 (85.2)	<0.001
Duration of MV, hours	0 [0–58.5]	0 [0–55]	23 [3.5–79.5]	<0.001
Therapies received in the PICU				
Inotropic score	0 [0-5]	0 [0–2.5]	0 [0-15]	<0.001
Inotrope, n	65 (32.2)	35 (24.8)	30 (49.2)	<0.001
Inotrope^b^, n	58 (28.7)	32 (22.7)	26 (42.6)	0.002
Steroid, n	140 (69.3)	94 (66.7)	46 (75.4)	0.247
Change in antibiotics, n	24 (11.9)	22 (15.6)	2 (3.3)	0.016
Furosemide, n	142 (70.3)	90 (63.8)	52 (85.2)	<0.001
Hemofiltration, n	12 (5.9)	7 (5.0)	5 (8.2)	0.372

Values are median [interquartile range]. Numbers in parentheses denote percentages. AKI, acute kidney injury; ALI, acute lung injury; DIC, disseminated intravascular coagulation; FO, fluid overload; MODS, multi-organ dysfunction syndrome; MV, mechanical ventilation; PICU, pediatric intensive care unit; PIM2, pediatric index of mortality 2; RRT, renal replacement therapy.

^a^Developed or administered during PICU stay.

^b^Administration during the first day after PICU admission.

P value, comparison between survivors and non-survivors.

### Fluid overload

Early fluid overload was analyzed in all patients (n = 202). The median fluid accumulation in the first 24 hours of admission was 1.32% (IQR -0.24%-4.07%). Among all the patients, 41 (20.3%) experienced early fluid overload (FO ≥5%) in the first 24 hours of PICU admission, including 9 (4.5%) with FO ≥10%. Early fluid overload was significantly positively correlated with fluid intake (r = 0.144, P = 0.040) and negatively correlated with fluid output (r = -0.402, P <0.001).

PICU-acquired daily fluid overload and the fluctuation in fluid accumulation were analyzed in patients who had at least 48 hours of PICU stay (n = 154). Forty-eight (23.8%) patients were excluded, including 28 (46%) non-survivors and 20 (14%) survivors (P <0.001). The median maximum daily fluid accumulation in patients with at least 48 hours of PICU stay during the 2 to 7 day period following admission was 3.48% (IQR 1.73%-4.83%). Among these patients, 36 (23.4%) developed PICU-acquired daily fluid overload (FO ≥5%), including 2 (1.0%) with FO ≥10%. PICU-acquired daily fluid overload was negatively correlated with fluid output (r = -0.278, P <0.001), but it was not correlated with fluid intake (r = 0.051, P = 0.534).

To evaluate whether the largest change in fluid balance during the first 7 days after PICU admission was associated with adverse outcomes, the fluctuation in fluid accumulation was defined. The median fluctuation in fluid accumulation during the first 7 days of PICU stay was 4.96% (IQR 3.17%-7.87%) in patients with at least 48 hours of PICU stay (n = 154). Among these patients, 77 (50.0%) had fluctuations in fluid accumulation <5%, 59 (38.3%) had fluctuations between 5% and 10%, and 18 (11.7%) had fluctuations ≥10%.

**Fig 1:**
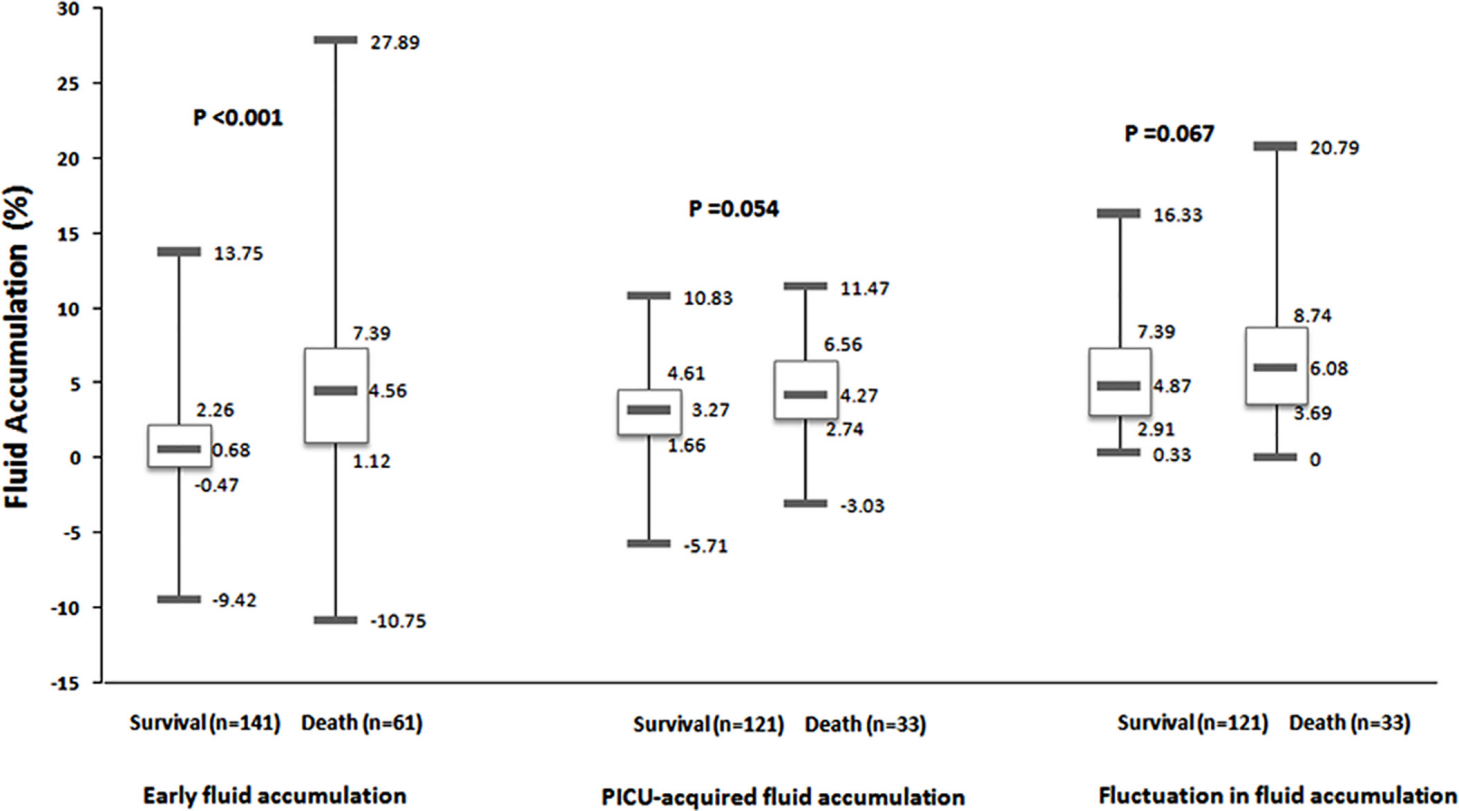
Comparison of fluid accumulation between survivors and non-survivors. Early fluid accumulation: occurring in the first 24 hours of PICU admission analyzed in all patients (n = 202). PICU-acquired daily fluid accumulation: analyzed the maximum daily fluid accumulation occurring during the next 6 days in patients with at least 48 hours of PICU stay (n = 154). Fluctuation in fluid accumulation: calculated as the difference between the maximum and the minimum daily fluid accumulation obtained during the first 7 days after PICU admission in patients with at least 48 hours of PICU stay (n = 154). Boxes represent medians and interquartile ranges and whiskers represent minimums and maximums. P-values refer to comparison of early fluid accumulation, PICU-acquired daily fluid accumulation or the fluctuation in fluid accumulation between survivors and non-survivors.

The early fluid accumulation, PICU-acquired daily fluid accumulation, and fluctuation in fluid accumulation between patients with severe sepsis who did and did not survive are compared in Fig 1. Comparisons of demographic and clinical characteristics and secondary outcomes between patients with and without early fluid overload and between patients with and without PICU-acquired daily fluid overload are summarized in Tables 2 and 3. The same comparison was also performed between patients with fluctuations in fluid accumulation <10% and ≥10%; however, none met statistical significance (data not shown).

**Table 2:** Comparison of demographic and clinical characteristics between severe septic patients with and without fluid overload.

	Early fluid overload			PICU-acquired daily fluid overload		
	<5% (n = 161)	≥5% (n = 41)	P	<5% (n = 118)	≥5% (n = 36)	P
Age, yeas	0.5 [0.2–1.6]	0.5 [0.3–1.1]	0.993	0.5 [0.2–1.5]	0.5 [0.2–1.1]	0.345
Body weight, kg	8 [5.5–11]	7.5 [5.5–9.3]	0.318	7.5 [5.5–10.1]	7.1 [4.6–9.0]	0.123
Male, n	82 (50.9)	25 (61)	0.250	60 (50.8)	21 (58.3)	0.431
PIM2	3.3 [2–15.8]	16.2 [4.6–52.7]	0.000	3.2 [2.0–12.2]	5.1 [2.7–22.7]	0.156
Fluid intake, L	0.72 [0.05–1.14]	0.92 [0.63–1.63]	0.019	0.89 [0.69–1.30]	0.96 [0.79–1.41]	0.258
Fluid output, L	0.68 [0.42–1.12]	0.40 [0.07–0.79]	<0.001	0.73 [0.54–1.05]	0.64 [0.38–0.86]	0.122
Therapies received in the PICU						
Inotropic score	0 [0-5]	0 [0-10]	0.128	0 [0-5]	0 [0–3.75]	0.690
Inotrope, n	48 (29.8)	17 (41.4)	0.154	36 (30.5)	9 (25.0)	0.525
Steroid, n	114 (70.8)	26 (63.4)	0.360	89 (75.4)	28 (77.8)	0.772
Change in antibiotics, n	22 (13.7)	2 (4.9)	0.176	23 (19.5)	1 (2.8)	0.016
Furosemide, n	113 (70.2)	29 (70.7)	0.946	88 (74.6)	28 (77.8)	0.697
Hemofiltration, n	8 (5)	4 (9.8)	0.268	5 (4.2)	5 (13.9)	0.054
Therapies received prior to the PICU						
Inotrope, n	13 (8.1)	1 (2.4)	0.309	10 (8.5)	3 (8.3)	0.979
Steroid, n	30 (18.6)	9 (21.9)	0.631	21 (17.8)	7 (19.4)	0.822
Antibiotic, n	119 (73.9)	24 (58.5)	0.053	87 (73.7)	26 (72.2)	0.858

Values are median [interquartile range]. Numbers in parentheses denote percentages. NA, not applicable; PICU, pediatric intensive care unit; PIM2, pediatric index of mortality 2. Early fluid overload: fluid overload occurring in the first 24 hours of PICU admission analyzed in all patients. PICU-acquired daily fluid overload: the maximum daily fluid accumulation occurring during the next 6 days in patients with at least 48 hours of PICU stay.

**Table 3:** Comparison of secondary outcomes between severe septic patients with and without fluid overload.

	Early fluid overload			PICU-acquired daily fluid overload		
	<5% (n = 161)	≥5% (n = 41)	P	<5% (n = 118)	≥5% (n = 36)	P
AKI^a^, n	19 (11.8)	17 (41.5)	<0.001	15 (12.7)	11 (30.6)	0.012
Need for RRT^a^, n	21 (13.0)	19 (46.3)	<0.001	15 (12.7)	13 (36.1)	0.001
ALI^a^, n	56 (34.8)	29 (70.7)	<0.001	47 (39.8)	12 (33.3)	0.483
Shock^a^, n	37 (23)	22 (53.7)	<0.001	27 (22.9)	12 (33.3)	0.207
DIC^a^, n	13 (8.2)	12 (29.3)	<0.001	7 (5.9)	6 (16.7)	0.043
MODS^a^, n	31 (19.3)	21 (51.2)	<0.001	21 (17.8)	11 (30.6)	0.099
MV^a^, n	61 (37.9)	31 (75.6)	<0.001	51(43.2)	15 (41.7)	0.869
Duration of MV, hours	0 [0–60.5]	8 [0.5–38]	0.012	0 [0–91.5]	0 [0–126]	0.299
PICU LOS^b^, days	4 [2–9]	1 [1–5.5]	<0.001	5.5 [3–10]	5.5 [4–14]	0.445
PICU LOS^c^, days	5 [3–9.3]	5 [2–10]	0.602	6 [3–10]	6 [4–10.8]	0.469
Death, n	31 (19.3)	30 (73.2)	<0.001	21 (17.8)	12 (33.3)	0.047

Values are median [interquartile range]. Numbers in parentheses denote percentages. AKI, acute kidney injury; ALI, acute lung injury; DIC, disseminated intravascular coagulation; LOS, length of stay; MODS, multi-organ dysfunction syndrome; MV, mechanical ventilation; PICU, pediatric intensive care unit; RRT, renal replacement therapy. Early fluid overload: fluid overload occurring in the first 24 hours of PICU admission analyzed in all patients. PICU-acquired daily fluid overload: the maximum daily fluid accumulation occurring during the next 6 days in patients with at least 48 hours of PICU stay.

^a^Developed or administered during PICU stay.

^b^Length of stay of all patients.

^c^Length of stay of survivors.

### Association of fluid overload with mortality

Early fluid overload (OR = 1.22; 95% CI 1.12–1.33; P <0.001; n = 202) and fluid intake occurring in the first 24 hours of PICU admission (OR = 1.59; 95% CI 1.19–2.13; P = 0.002), but not fluid output (OR = 1.23; 95% CI 0.96–1.61; P = 0.097), were significantly associated with PICU mortality. The association of early fluid overload (AOR = 1.25; 95% CI 1.13–1.39; P <0.001), but not fluid intake (AOR = 1.32; 95% CI 0.95–1.85; P = 0.101), with PICU mortality remained significant after adjustment for age and illness severity as assessed by PIM2 score.

Both PICU-acquired daily fluid overload (OR = 1.16; 95% CI 1.00–1.34; P = 0.048; n = 154) and fluid intake occurring during the same day (OR = 1.65; 95% CI 1.07–2.55; P = 0.025), but not fluid output (OR = 1.40; 95% CI 0.90–2.19; P = 0.137), were significantly associated with PICU mortality. The association of PICU-acquired daily fluid overload (AOR = 1.19; 95% CI 1.02–1.40; P = 0.027), but not fluid intake (AOR = 1.43; 95% CI 0.91–2.23; P = 0.122), with mortality remained significant after adjustment for age and PIM2 score.

The fluctuation in fluid accumulation, which was defined as the difference between the maximum and the minimum daily fluid accumulation obtained during the first 7 days after PICU admission, was associated with mortality (OR = 1.11; 95% CI 1.01–1.22; P = 0.032; n = 154). However, the association did not remain significant after controlling for age and PIM2 score (AOR = 1.10; 95% CI 0.99–1.23; P = 0.063).

**Fig 2:**
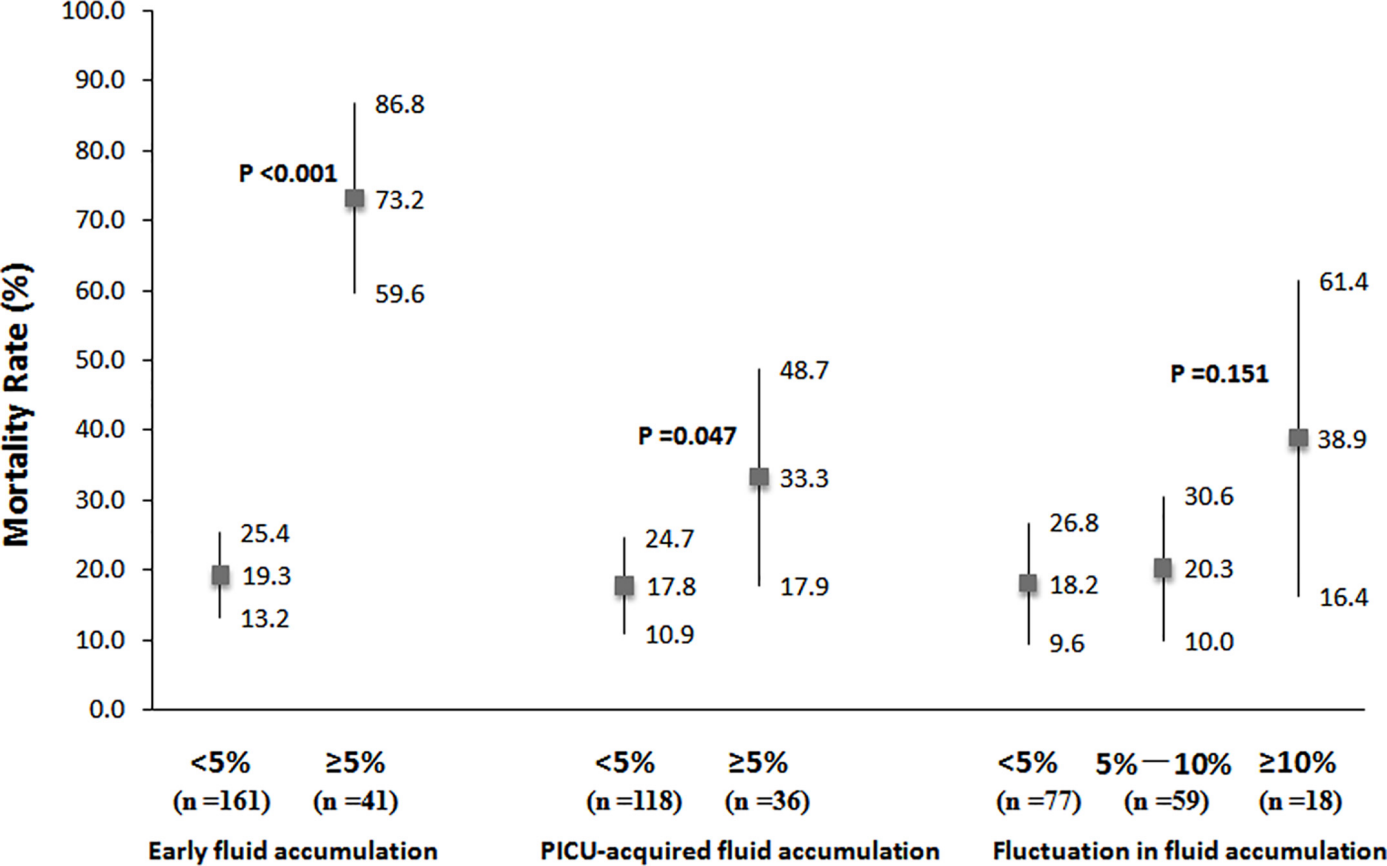
Comparison of mortality rates between severe septic patients with and without fluid overload. Early fluid accumulation: occurring in the first 24 hours of PICU admission analyzed in all patients (n = 202). PICU-acquired daily fluid accumulation: analyzed the maximum daily fluid accumulation occurring during the next 6 days in patients with at least 48 hours of PICU stay (n = 154). Fluctuation in fluid accumulation: calculated as the difference between the maximum and the minimum daily fluid accumulation obtained during the first 7 days after PICU admission in patients with at least 48 hours of PICU stay (n = 154). Error bars represent mortality rate and 95% confidence interval. P values refer to comparison between patients with early fluid accumulation <5% and ≥5%, between patients with acquired fluid accumulation <5% and ≥5%, and among patients with fluctuations in fluid accumulation <5%, 5%-10%, and ≥10%.

Comparisons of mortality rate between patients with and without early fluid overload, between patients with and without PICU-acquired daily fluid overload, and among patients with fluctuations in fluid accumulation<5%, 5%-10%, and ≥10% are displayed in Fig 2. In addition, since a total of 48 (23.8%) patients with less than a 48-hour PICU stay were excluded from the analyses of PICU-acquired daily fluid overload and fluctuation in fluid accumulation, comparisons of net fluid accumulation at 48 hours after PICU admission between survivors and non-survivors are shown in S1 Fig.

### Multivariate regression analysis of variables potentially associated with PICU mortality

To identify whether fluid overload was independently associated with increased risk of death in patients with severe sepsis, variables with p <0.05 in the comparison of Table 1, considered as confounding factors, were entered into multivariable binary logistic regression analysis with the backward method after excluding multicollinear variables. Collinearity diagnostics confirmed the absence of significant multicollinearity among independent variables (data not shown).

For early fluid overload analysis (n = 202), the final model retained age (AOR = 1.21; 95% CI 1.06–1.38; P = 0.005), MODS (AOR = 2.72; 95% CI 1.16–6.68; P = 0.029), mechanical ventilation (AOR = 10.11; 95% CI 4.01–25.52; P <0.001), change in antibiotics (AOR = 0.15; 95% CI 0.03–0.80; P = 0.026), and early fluid overload (AOR = 1.20; 95% CI 1.08–1.33; P <0.001) as significant factors associated with mortality, as shown in Table 4.

**Table 4:** Multivariate logistic regression analysis of early fluid overload and confounding variables associated with PICU mortality.

	AOR	95% CI	P
Age	1.21	1.06–1.38	0.005
MODS	2.72	1.11–6.68	0.029
MV	10.11	4.01–25.52	<0.001
Change in antibiotics	0.15	0.03–0.80	0.026
Early FO	1.20	1.08–1.33	0.001

AOR, adjusted odds ratio; CI, confidence interval; FO, fluid overload; MODS, multi-organ dysfunction syndrome; MV, mechanical ventilation; PICU, pediatric intensive care unit. Variables of age, PIM2 score, AKI, ALI, Shock, DIC, MODS, change in antibiotics, and the use of MV, inotropes, and furosemide were entered into the multivariate logistic regression analysis with the backward method, as confounding factors. Early fluid overload was coded as a continuous variable (n = 202). The P value for the Hosmer-Lemeshow goodness-of-fit test was 0.140.

For the analysis of PICU-acquired daily fluid overload (n = 154), backward logistic regression analysis showed that PICU-acquired daily fluid overload was an independent risk factor associated with PICU mortality (AOR = 5.47 per log increase; 95% CI 1.15–25.96; P = 0.032), as displayed in Table 5. For the analysis of the fluctuation in fluid accumulation, the final model only retained mechanical ventilation as an independent risk factor associated with PICU mortality (AOR = 10.19; 95% CI 3.84–27.06; P <0.001; n = 154).

**Table 5:** Multivariate logistic regression analysis of PICU-acquired daily fluid overload and confounding variables associated with PICU mortality.

	AOR	95% CI	P
Age^a^	2.56^b^	1.12–5.88	0.026
MV	7.86	2.88–21.46	<0.001
PICU-acquired FO^a^	5.47^c^	1.15–25.96	0.032

AOR, adjusted odds ratio; CI, confidence interval; FO, fluid overload; MV, mechanical ventilation; PICU, pediatric intensive care unit. Variables of age, PIM2 score, AKI, ALI, Shock, DIC, MODS, change in antibiotics, and the use of MV, inotropes, and furosemide were entered into the multivariate logistic regression analysis with the backward method, as confounding factors. PICU-acquired daily fluid overload was coded as a continuous variable (n = 154).

^a^Data were log-transformed to fit model assumptions.

^b^AOR represents the increase in odds per log increase in age.

^c^AOR represents the increase in odds per log increase in FO.

The P value for the Hosmer-Lemeshow goodness-of-fit test was 0.507.

### Ability of fluid overload to predict PICU mortality

**Fig 3:**
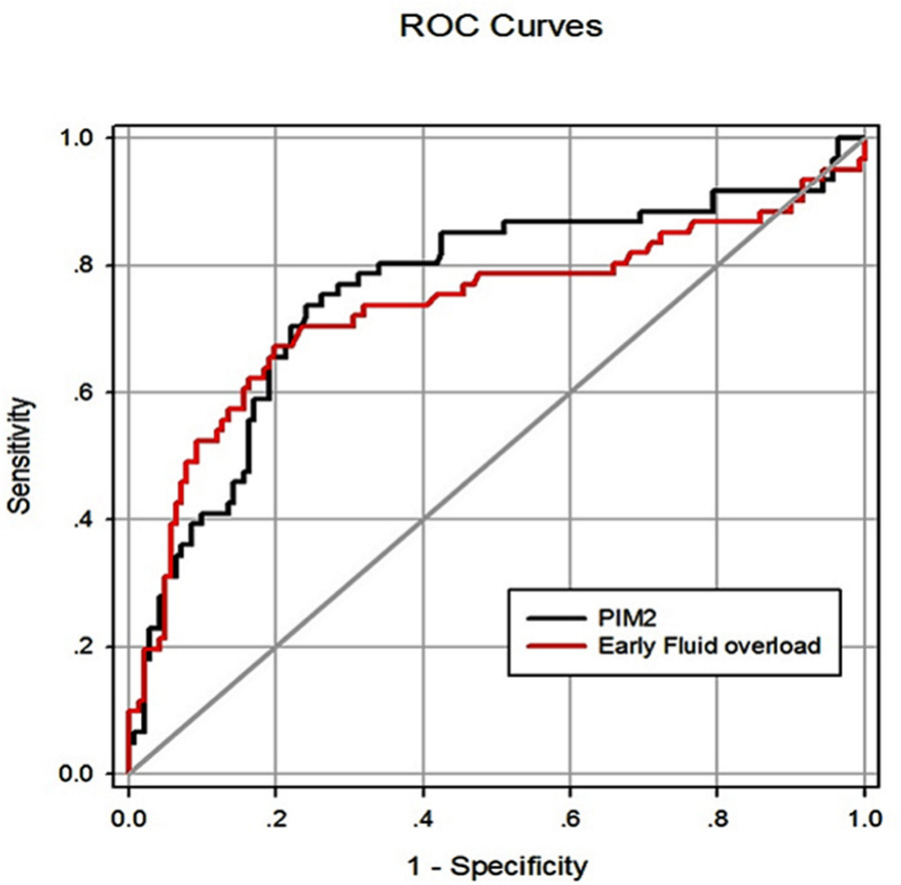
ROC curves for the ability of early fluid overload and PIM2 to predict PICU mortality in patients with severe sepsis. AUC, the area under the ROC curve; PIM2, pediatric index of mortality 2; ROC, Receiver operating characteristic. The AUCs for early fluid overload and PIM2 were 0.74 and 0.76, respectively (n = 202). The P value for the comparison of the difference between the AUCs is 0.656.

The performances of fluid overload and confounding variables in predicting PICU mortality are assessed in Table 6. Early fluid overload as a continuous variable, but not PICU-acquired daily fluid overload or the fluctuation in fluid accumulation, was predictive of PICU mortality and achieved an AUC of 0.74 (95% CI 0.65–0.82; P <0.001; n = 202), which was similar to the result obtained based on the PIM2 score (AUC = 0.76; 95% CI 0.68–0.84; P <0.001; n = 202), as shown in Fig 3. The sensitivity and specificity for early fluid overload at the optimal cut-off value of 2.65% in predicting PICU mortality in all patients with severe sepsis were 67.2% and 80.1%, respectively.

**Table 6:** Predictive performance of fluid overload and confounding variables for PICU mortality.

	AUC	95% CI	P	Optimal cut-off value	Sensitivity (%)	Specificity (%)
Age^a^	0.62	0.53–0.70	0.008	NA	NA	NA
PIM2^a^	0.76	0.68–0.84	<0.001	8.2	73.8	75.9
Early FO^a^^,^^c^	0.74	0.65–0.82	<0.001	2.65	67.2	80.1
PICU-acquired FO^b^^,^^c^	0.61	0.49–0.73	0.054	4.74	45.5	77.7
Fluctuation in FO^b^^,^^c^	0.60	0.50–0.71	0.067	2.91	93.9	24.8

AUC, area under the-receiver-operating-characteristic curve; CI, confidence interval; FO, fluid overload; NA, not applicable; PICU, pediatric intensive care unit; PIM2, pediatric index of mortality 2.

^a^Analyzed in all patients (n = 202).

^b^Analyzed in patients with PICU length of stay **≥**48 hours (n = 154).

^c^Fluid overload was coded as a continuous variable.

### Association of fluid overload with secondary outcomes

To identify whether fluid overload, coded as a continuous variable, was independently associated with secondary outcomes in patients with severe sepsis, variables that were related to the outcome in univariate analysis were entered into the multivariable binary logistic regression analysis as confounding factors. Early fluid overload (n = 202) was significantly associated with AKI after adjustment for PIM2 score, ALI, DIC, MODS, shock, and the use of mechanical ventilation, inotropes, and furosemide (AOR = 1.15; 95% CI 1.03–1.28; P = 0.009), with shock after adjustment for PIM2 score, AKI, ALI, DIC, MODS, mechanical ventilation, inotropes, and furosemide (AOR = 1.19; 95% CI 1.07–1.32; P = 0.002), with DIC after adjustment for age, PIM2 score, AKI, ALI, MODS, shock, mechanical ventilation, and inotrope (AOR = 1.14; 95% CI 1.02–1.26; P = 0.018), and with the use of mechanical ventilation after adjustment for age, PIM2 score, AKI, ALI, DIC, MODS, shock, inotropes, and furosemide (AOR = 1.20; 95% CI 1.01–1.42; P = 0.042). In contrast, early fluid overload was not associated with ALI and MODS after multivariable adjustment. Early fluid overload was also not associated with the duration of mechanical ventilation or the PICU length of stay even in univariate analysis (data not shown).

The association of PICU-acquired daily fluid overload and AKI remained significant after adjustment for PIM2 score, ALI, DIC, MODS, shock, and the use of mechanical ventilation, inotropes, and furosemide (AOR = 1.29; 95% CI 1.03–1.63; P = 0.027; n = 154). The fluctuation in fluid accumulation of ≥10% during the first 7 days of PICU stay remained significantly associated with shock (AOR = 1.16; 95% CI 1.01–1.33; P = 0.036) and MODS (AOR = 1.19; 95% CI 1.02–1.40; P = 0.031), but not with AKI, ALI, DIC, and the use of mechanical ventilation after adjustment for the confounding factors.

In addition, the PICU length of stay increased with greater fluctuations in fluid accumulation in all patients with at least a 48-hour PICU stay (FO <5%, 5%-10% vs. ≥10%: 4 [3–8], 7 [4–11] vs. 10 [6–16] days; P <0.001; n = 154) and in survivors (FO <5%, 5%-10% vs. ≥10%: 4 [3–8], 7 [5–11] vs. 10 [5–15] days; P <0.001; n = 121).

## Discussion

Our study provides data on fluid accumulation in critically ill children with severe sepsis and demonstrates that both early and PICU-acquired fluid overload were independently associated with PICU mortality, even after adjustment for illness severity.

The association between increasing fluid balance and worsening clinical outcomes found in our study is similar to findings from previous clinical studies conducted in adult patients with severe sepsis [22–24]. As reported by Boyd and colleagues, positive fluid balance both early in resuscitation and cumulatively over 4 days during an ICU stay is associated with an increased risk of mortality in adult patients with septic shock [23]. When analyzing PICU-acquired daily fluid overload, we studied a group of patients with at least 48 hours of PICU stay, who might have potentially benefited from appropriate early fluid management. Our data indicate that daily fluid overload was independently associated with a higher risk of mortality, regardless of the time of onset.

Patients with a high PIM2 score were prone to have early fluid overload in our study. This raises the question of whether it is possible that the greater PIM2 scores and greater numbers of patients with MODS, shock and DIC in the non-survivor group signified much sicker patients, which may have led to increased mortality rather than mortality being directly related to fluid overload. Due to the fact that this is a retrospective study, it is not possible to distinguish whether progressive fluid overload is simply a marker of illness severity or a causative factor of outcome. However, our study does demonstrate that both early and acquired daily fluid overload were independently associated with mortality even after controlling for severity of illness, as assessed by PIM2 score, and other potential confounders, such as the presence of AKI, MODS, shock, or DIC, in multivariate models. Notably, whether fluid overload is preventable or a consequence of illness severity is an important aspect to fluid management. Unlike early fluid overload, PICU-acquired fluid overload was not related to illness severity and the presence of MODS and shock, showing that PICU-acquired fluid overload might be preventable. Our data imply that PICU mortality might be negatively impacted by fluid overload regardless of the presence of AKI, MODS, shock, and DIC, suggesting that interventions to limit the development of a positive cumulative fluid balance may be associated with improved clinical outcomes in children with severe sepsis.

Although there is increasing awareness of the impact of fluid overload on clinical outcomes, there remains no consensus regarding criteria for defining fluid accumulation in pediatric populations. Most previous pediatric studies have used the adult standard defined as fluid accumulation >10% over baseline [11,19,21,37]. However, Hassinger et al. defined early fluid overload as a fluid balance 5% above body weight [17]. This threshold was recommended as a cutoff point for higher AKI risk in patients who require mechanical ventilation and one or more vasoactive agents [17,32]. In our study, the percentage of septic children with FO ≥10% during the first 7 days after PICU admission was approximately 5.0%. The number of pediatric patients identified as having FO >10% has varied in different studies [11,31,37]. The reason for the low incidence of fluid overload in our critical care setting might be explained by our therapy policy, which advocates more restrictive fluid management and early implementation of diuretics and inotropes. In our study, 70.3% of the patients received furosemide therapy during their PICU stay. Thus, we set a fluid accumulation of ≥5% as the threshold for defining early and acquired daily fluid overload. The finding of an association between early and acquired daily fluid overload and mortality in our study offers the potential of using a FO of 5% as a threshold for possible interventions in critically ill children with severe sepsis.

The other major finding in this study was the association between fluid overload and secondary outcomes according to multivariable logistic regression analysis. After multivariable adjustment, both early and PICU-acquired daily fluid overload were significantly associated with AKI developed during PICU stay. Previous studies have suggested that significant fluid overload may occur prior to the development of AKI [17,38]. However, in our study, among the 23 patients who developed both fluid overload and AKI during the first 7 days after PICU admission, only 3 patients occurred fluid overload prior to the diagnosis of AKI. It may not be surprising that AKI patients are more likely to develop fluid overload. In addition, fluid accumulation was more related to fluid output and less related to fluid intake in our study, indicating that the observed PICU mortality may have been more related to delayed removal of fluid. Our results suggest that patients may have benefited from more aggressive removal of fluid via medication or hemofiltration.

Fluid administration to critically ill patients is essential and lifesaving, and early and aggressive fluid resuscitation has been recommended for critically ill patients with severe sepsis and septic shock [4,6,7]. However, the debate is still open. Some important considerations indicate that aggressive fluid resuscitation on ICU admission is a potential source of excessive fluid administration, which may lead to fluid overload and adverse outcomes [8,22,39,40]. In our study, both fluid intake and fluid accumulation were associated with mortality. However, after multivariable adjustment, early and PICU-acquired daily fluid overload, but not fluid administration, were associated with an increased risk of death in the PICU. Therefore, further randomized clinical trials to explore whether interventions aimed at reducing fluid administration/fluid accumulation may be linked with improved outcomes are needed in children with severe sepsis.

In this study, we also found that patients who had a greater degree of fluctuation in fluid accumulation during the first 7 days of PICU stay had a higher rate of mortality and experienced a significantly longer PICU stay. Information on the relationship between fluctuations in fluid accumulation and clinical outcomes is limited. To the best of our knowledge, ours is the first study to explore the possible influence of fluctuations in fluid accumulation in patients with severe sepsis. We stratified patients by degree of fluctuation in fluid overload into 3 groups: <5%, 5%-10%, and ≥10% fluid overload. However, a greater fluctuation in fluid accumulation was not independently associated with an increased risk of mortality in our study. Unlike fluctuations in fluid accumulation and although all patients with an early fluid overload of ≥5% had a shorter PICU stay, neither early fluid overload nor PICU-acquired daily fluid overload were associated with overall length of stay in the PICU. The short PICU stay experienced by the patients with early fluid overload was because non-surviving patients had a shorter PICU stay, and the majority of patients (73.2%) with an early FO ≥5% died during their PICU stay.

This study has some limitations. First, we were not able to include fluid intake and output prior to PICU admission due to practical constraints. This might have resulted in an underestimation of fluid overload, as many patients would likely have received large volumes of fluid resuscitation prior to PICU admission [41]. Second, PICU-acquired daily fluid overload was only analyzed in patients who had at least 48 hours of PICU stay. A significantly higher number of patients were excluded in the non-survivor group compared to the survivor group. It is possible that the exclusion of patients who stayed in the PICU for less than 48 hours could have significantly weakened the power of the corresponding analyses. Third, our study population is younger than that usually reported in previous studies. However, the data support that severe sepsis is more prevalent in neonates and infants [1]. The positive correlation between age and mortality in this study might have been due to the higher prevalence of severe underlying diseases or comorbidities, such as respiratory and neurological problems, in older children rather than because of a direct effect of age [42,43]. Fourth, the mortality rate for patients with severe sepsis in our study was 30.2%, which was higher than that reported in a U.S. cohort, at 21.2% [44] and in an international cohort (the Sepsis PRevalence, OUtcomes, and Therapies study), at 23.0% [45]. There are quite a number of factors that may affect the outcome of patients with severe sepsis, such as the severity of illness, the administration of effective antibiotics, the timing and type of fluid resuscitation, and the use of vasopressors/inotropes [9,46]. Although most patients (70.3%) received diuretics in our study, few patients received hemofiltration (5.9%), and only 32.2% of the patients received inotropic agents during their PICU stay. Fifth, our results might be biased by the fact that we conducted a single-center retrospective observational study. Whether this information could inform future iterations of sepsis guidelines as to the safe volume for fluid resuscitation, further large multicenter prospective studies evaluating the effect of fluid balance on clinical outcomes in children with severe sepsis are required to confirm our findings.

## Conclusions

Our study indicates that both early and PICU-acquired daily fluid overload were independently associated with PICU mortality in children with severe sepsis. Further randomized clinical studies are needed to determine whether interventions to limit the development of fluid overload in critically ill children with severe sepsis are associated with improved outcomes.

## Supporting Information

S1 Fig Comparison of net fluid accumulation at 48 hours after PICU admission between survivors and non-survivors. A: Comparison between survivors and non-survivors who died within the first 48 hours of PICU admission. B: Comparison between survivors and non-survivors who died during their PICU stay. Boxes represent medians and interquartile ranges and whiskers represent minimums and maximums. P-values refer to comparison of net fluid accumulation between survivors and non-survivors.
(PPTX)

S1 Text Formula of Net fluid accumulation. (DOCX)

## Abbreviations

AKI: acute kidney injury
ALI: acute lung injury
AOR: adjusted odds ratio
CI: confidence interval
Cr: creatinine
DIC: disseminated intravascular coagulation
EGDT: early goal-directed therapy
FO: fluid overload
IQR: interquartile range
LOS: length of stay
MODS: multi-organ dysfunction syndrome
MV: mechanical ventilation
RRT: renal replacement therapy
OR: odds ratio
PICU: pediatric intensive care unit
PIM2: pediatric index of mortality 2
