# Supplementary figures and images for: Association of Fluid Accumulation with Clinical Outcomes in Critically Ill Children with Severe Sepsis
Source: PLoS One. 2016 Jul 28;11(7):e0160093. doi: 10.1371/journal.pone.0160093 (PMC4965086; doi:10.1371/journal.pone.0160093)

## Slide 1
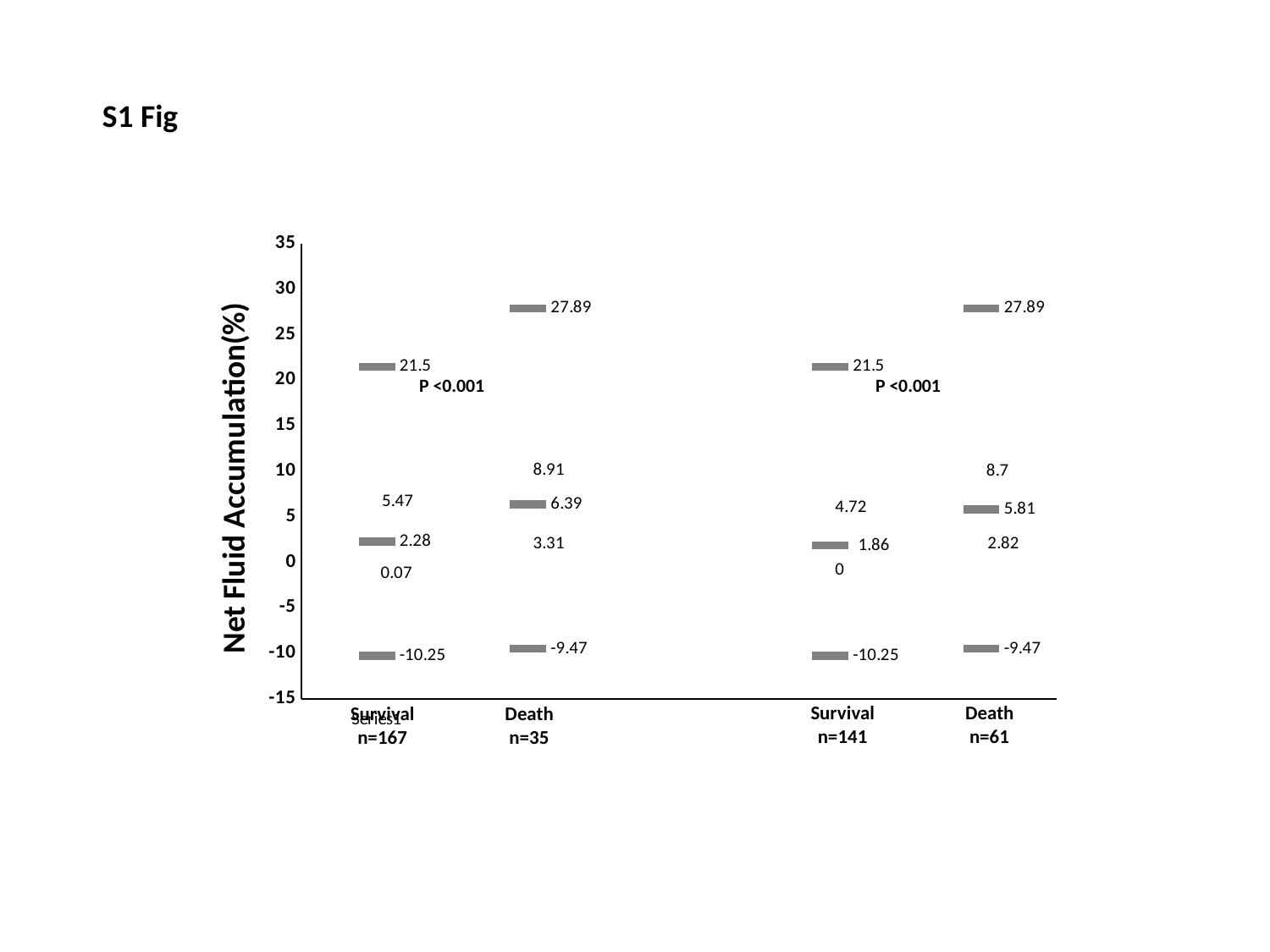

S1 Fig
### Chart
| Category | | | | | |
|---|---|---|---|---|---|
| | 0.07000000000000003 | -10.25 | 21.5 | 2.28 | 5.470000000000002 |
| | 3.3099999999999987 | -9.47 | 27.89 | 6.39 | 8.91 |
| | None | None | None | None | None |
| | 0.0 | -10.25 | 21.5 | 1.86 | 4.72 |
| | 2.82 | -9.47 | 27.89 | 5.81 | 8.700000000000001 |P <0.001
P <0.001
Survival
n=141
Death
n=61
Survival
n=167
Death
n=35

Supplement: S1 Fig — A: Comparison between survivors and non-survivors who died within the first 48 hours of PICU admission. B: Comparison between survivors and non-survivors who died during their PICU stay. Boxes represent medians and interquartile ranges and whiskers represent minimums and maximums. P-values refer to comparison of net fluid accumulation between survivors and non-survivors. (PPTX) [file pone.0160093.s001.pptx]
