# Supplementary material for: Association of Fluid Accumulation with Clinical Outcomes in Critically Ill Children with Severe Sepsis
Source: PLoS One. 2016 Jul 28;11(7):e0160093. doi: 10.1371/journal.pone.0160093 (PMC4965086; doi:10.1371/journal.pone.0160093)
Supplement: S1 Text — (DOCX) [file pone.0160093.s002.docx]

**Supporting Information**

**S1 Text:** Net fluid accumulation, expressing the cumulative FO% from the day of PICU admission to the given study day, was calculated as follows: [cumulative fluid intake from admission to the given study day (L) - cumulative fluid output from admission to the given study day (L)] / PICU admission weight (Kg) × 100% [19]. Net fluid accumulation at 48 hours after PICU admission was calculated as follows: [cumulative fluid intake from admission to 48 hours after PICU admission (L) - cumulative fluid output from admission to 48 hours after PICU admission (L)] / PICU admission weight (Kg) × 100%.
